# Decreasing Intracellular Entropy by Increasing Mitochondrial Efficiency and Reducing ROS Formation—The Effect on the Ageing Process and Age-Related Damage

**DOI:** 10.3390/ijms25126321

**Published:** 2024-06-07

**Authors:** Borut Poljšak, Irina Milisav

**Affiliations:** 1 Laboratory of Oxidative Stress Research, Faculty of Health Sciences, University of Ljubljana, Zdravstvena pot 5, SI-1000 Ljubljana, Slovenia; borut.poljsak@zf.uni-lj.si; 2 Faculty of Medicine, Institute of Pathophysiology, University of Ljubljana, Zaloska 4, SI-1000 Ljubljana, Slovenia

**Keywords:** entropy, disorder, randomness, ageing, mitochondrial function, energy efficiency

## Abstract

A hypothesis is presented to explain how the ageing process might be influenced by optimizing mitochondrial efficiency to reduce intracellular entropy. Research-based quantifications of entropy are scarce. Non-equilibrium metabolic reactions and compartmentalization were found to contribute most to lowering entropy in the cells. Like the cells, mitochondria are thermodynamically open systems exchanging matter and energy with their surroundings—the rest of the cell. Based on the calculations from cancer cells, glycolysis was reported to produce less entropy than mitochondrial oxidative phosphorylation. However, these estimations depended on the CO_2_ concentration so that at slightly increased CO_2_, it was oxidative phosphorylation that produced less entropy. Also, the thermodynamic efficiency of mitochondrial respiratory complexes varies depending on the respiratory state and oxidant/antioxidant balance. Therefore, in spite of long-standing theoretical and practical efforts, more measurements, also in isolated mitochondria, with intact and suboptimal respiration, are needed to resolve the issue. Entropy increases in ageing while mitochondrial efficiency of energy conversion, quality control, and turnover mechanisms deteriorate. Optimally functioning mitochondria are necessary to meet energy demands for cellular defence and repair processes to attenuate ageing. The intuitive approach of simply supplying more metabolic fuels (more nutrients) often has the opposite effect, namely a decrease in energy production in the case of nutrient overload. Excessive nutrient intake and obesity accelerate ageing, while calorie restriction without malnutrition can prolong life. Balanced nutrient intake adapted to needs/activity-based high ATP requirement increases mitochondrial respiratory efficiency and leads to multiple alterations in gene expression and metabolic adaptations. Therefore, rather than overfeeding, it is necessary to fine-tune energy production by optimizing mitochondrial function and reducing oxidative stress; the evidence is discussed in this paper.

## 1. Introduction

Entropy is a thermodynamic property that quantifies the degree of disorder or randomness in a system. In simple terms, it measures the number of ways the components of a system can be arranged without losing identical macroscopic properties. A system that has high entropy is more disordered and has more possible arrangements, while a system with low entropy is more ordered and has fewer possible arrangements. Entropy, also a measure of the chaos or uncertainty of a system [1], tends to increase in isolated systems according to the second law of thermodynamics. “Living systems are open systems, maintaining themselves in exchange of materials with the environment, and in continuous building up and breaking down of their components” [2]. Living organisms are inherently out of equilibrium [3] due to a constant consumption and dissipation of energy [4]. Living organisms, such as humans and other mammals, must consume energy sources all the time. The energy produced by the oxidation of organic substances such as carbohydrates, fats, and amino acids is needed by organisms to maintain body composition and temperature, engage in physical work, and synthesize, transport, as well as replace damaged molecules [5]. A living organism is highly ordered with low entropy, because of its interactions with the environment. While entropy cannot be reduced in the overall system, cells can maintain local order by actively controlling and reducing entropy within their boundaries. Although organisms increase their degree of organization—and thus decrease entropy—by growing, evolving, and becoming more complex, they do so at the expense of energy and matter exchange, which increases entropy in their environment [6]. However, this order comes at a cost, and large amounts of energy must be invested to maintain the organism’s low entropy. If entropy is applied at the cellular level, a state of disorder means the deterioration of the physical structures of macromolecules such as proteins, DNA, and membranes [7,8], leading to an absence of orderliness at the cellular, subcellular, proteomic, transcriptomic, and genomic levels [9]. At the cellular level, maintaining a certain level of organization is essential for proper functioning. In line with the second law of thermodynamics, ageing leads to a transition on different time scales from metabolic reactions at a state of greater disequilibrium to a state of less disequilibrium (with higher entropy) [10]. The total entropy of an organism increases in ageing due to modified cellular and molecular processes. The accumulation of molecular damage, cellular dysfunction, and a loss of biological complexity over time contribute to an increase in disorder and randomness in the organism, thus resulting in higher entropy. ATP production becomes insufficient during ageing [11]. Therefore, energy for DNA repair and cellular maintenance processes is limited and metabolism is reprogrammed [12]. The question is how to efficiently increase the energy availability, which is needed for better maintenance and repair and the antioxidant defence systems for a healthier and longer lifespan. One of the most important reasons for inadequate defense against ROS-induced damage and poor functioning of cellular repair mechanisms is the high cost of energy expenditure. Kowald and Kirkwood projected that immortality could be practically attained if 55% of the total energy of the simulated cell was dedicated to restoring and/or preventing free radical and oxidative damage [13,14]. Therefore, the mechanisms that guard cells from oxidative stress (e.g., endogenous antioxidants and DNA repair processes) expend substantial energy when constantly activated in all cell compartments. It is the trade-off in apportioning (less) energy to the repair mechanisms that contribute to the gradual decline of the body with age [15,16,17,18]. If an external source induces a decrease in entropy, a counterclockwise effect is theoretically expected, leading to regenerative processes and/or an increase in the functional reserve that supports regenerative tissue/cell changes [19]. Therefore, longevity and health can be enhanced through the control of cellular repair and maintenance mechanisms important for reversing entropy by providing sufficient cellular energy for optimal operation of cellular repair and redox balance.

What are the possible clinical and lifestyle strategies to delay entropy or decrease the disruption of order processes? Methods and approaches to influence energy production by optimizing mitochondrial function will be presented. It should be noted, however, that not all aspects of ageing can be directly explained by the concept of entropy. The ageing process is influenced by numerous factors, including genetics, lifestyle, environmental factors, and stochastic events that cannot be fully captured by thermodynamic principles alone. The relation between ageing and the increase of entropy as the result of various degenerative processes was extensively studied [20,21,22,23,24,25,26,27,28]. The focus of this paper is on methods to reduce intracellular entropy by increasing mitochondrial function and energy efficiency, improving intracellular repair and maintenance systems, reducing ROS formation and cellular damage, and how this affects ageing.

**Figure 1:**
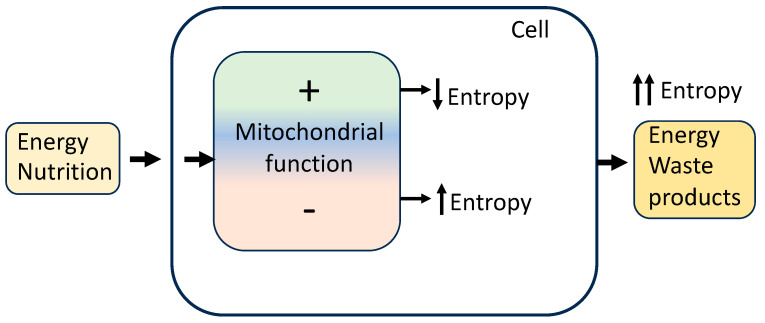
Organisms, cells, and mitochondria receive energy from an environment to reduce intracellular entropy by increasing mitochondrial function and energy conversion efficiency, improving repair and maintenance systems, and reducing ROS formation and cellular damage. These processes delay ageing. In contrast, decreased efficiency of energy conversion and decreased ability for cellular maintenance and repair lead to an increase in disorder and randomness (entropy), resulting in cell deterioration and accelerated ageing.

Organisms are not isolated systems; they are highly ordered and structured, and appear to reduce their internal entropy by creating well-ordered structures and other living processes at the expense of discharging higher entropy wastes (Figure 1). Nevertheless, the total entropy of living organisms and their environment increases.

## 2. Increasing Mitochondrial Efficiency

In redox regulation, the requirement of continuous monitoring of inflow and outflow to minimize deviation from the steady-state set point in the open metabolic system refers to physiological oxidative stress or oxidative eustress [29,30]. Such redox eustress and the feedback restoration of a steady state regulate the active maintenance of physiological health [31].

Mitochondrial efficiency refers to the ability of mitochondria to produce energy (ATP) while minimizing the production of reactive oxygen species (ROS), which may result in cellular damage. Mitochondrial efficiency is influenced by factors such as the balance between energy production and consumption, the electron transport chain, and the function of antioxidant systems within the cell. 

Mitochondrial DNA deletions and mitochondrial DNA point mutations accumulate with age in organisms ranging from worms to humans [32,33,34]. In older organisms, the mitochondria become less numerous, larger, and not as efficient because they produce less energy and more ROS. Older mitochondria, therefore, produce less energy that can be used for optimal damage repair, resulting in heightened oxidative damage to DNA, lipids, and proteins, leading to increased entropy over time. Living systems are able to reverse the principle of entropy increase under conditions of increased energy supply, e.g., by increased mitochondrial efficiency, nutrient supply that provides compounds that affect the Krebs cycle and oxidative phosphorylation, an increase in cellular defence and protection systems, etc. This is because an organism or cell can interact with its environment through the exchange of energy, matter, and information to achieve lower disorder or “negative entropy”, which counteracts the increase in entropy; at the same time, it may release the generated entropy from its body/cell into the environment [8]. Increased mitochondrial efficiency can lead to decreased ROS formation, improved cellular function, and energy production and utilization that can be used for optimal cellular repair and maintenance systems, potentially having a positive impact on entropy as well as on overall health and ageing. All cellular systems involved in damage defence, maintenance, and repair at least indirectly reduce disorder and entropy in the cell. Below are discussed some strategies that have been explored to increase mitochondrial performance efficiency.

### 2.1. The Adequate Supply of Compounds Affecting the Krebs Cycle and Oxidative Phosphorylation

**Figure 2:**
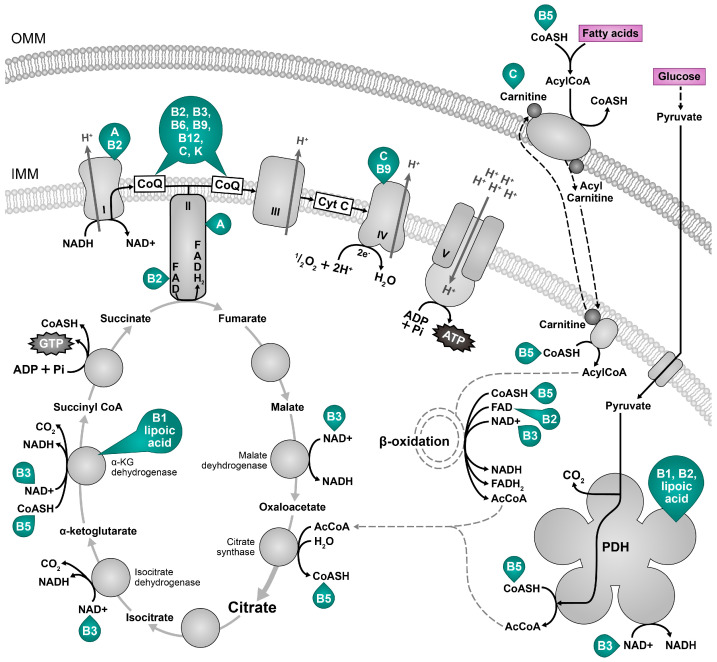
Nutrients for cellular energy generation. The main metabolic fuels, pyruvate from glucose and fatty acids (magenta) are terminally oxidized in the Krebs cycle in mitochondria to yield the reducing equivalents NADH and FADH2, which donate electrons to the electron transport chain that maintains a proton gradient necessary for the ATP synthesis. Vitamins A, B, C, K, E and lipoic acid are necessary for mitochondrial energy production and are depicted in green next to the resulting cofactor or molecule [52]; vitamin B7 (biotin) is necessary for fatty acid oxidation [53]; vitamin B9 (folic acid) is also required for mitochondrial DNA integrity—its deficiency can interfere with the gene expression of mitochondrially encoded transport chain components [77]. The oxidized form of vitamin E interferes with the activity of the respiratory chain complexes I (CI), II (CII), and III (CIII). B1 (thiamin), B2 (riboflavin), B3 (niacin), B5 (pantothenic acid), B6 (pyridoxine), B12 (cobalamin), C: vitamin C, K: vitamin K. α-KG dehydrogenase: alpha ketogutarate dehydrogenase, CoASH: coenzyme A, AcCoA: acetyl coenzyme A, CoQ: coenzyme Q, CI CIV: respiratory chain complexes I–IV, Cytc: cytochrome c, IMM: inner mitochondrial membrane, OMM: outer mitochondrial membrane, PDH: pyruvate dehydrogenase.

The Krebs cycle, known as the citric acid cycle or tricarboxylic acid cycle, and oxidative phosphorylation are two interrelated processes in the mitochondria of eukaryotic cells. Together, they generate adenosine triphosphate (ATP), the primary energy currency of cells. The Krebs cycle is a sequence of chemical reactions within the mitochondrial matrix in which acetyl-CoA (mainly generated during the breakdown of carbohydrates and fats) is oxidized to release carbon dioxide and electrons (Figure 2). ATP is then synthesized from ADP and inorganic phosphate (Pi), using the energy derived from electron transfer through the electron transport chain (ETC) in oxidative phosphorylation. The ETC is in the inner mitochondrial membrane and consists of several protein complexes (I, II, III, and IV) that transfer electrons from reduced forms of nicotinamide adenine dinucleotide (NADH) and flavin adenine dinucleotide (FADH_2_) to molecular oxygen (O_2_) (Figure 2). There are no consistent data supporting the benefits of the intake of large amounts of macronutrients (carbohydrates and fats), whereas the intake of specific micronutrients could affect mitochondrial function, e.g., the intake of foods containing molecules essential for the proper functioning of respiratory enzymes could help maintain mitochondrial performance. The protective effect of micronutrients targeting mitochondria is very promising for the treatment of age-related and metabolic diseases. Wesseling and colleagues reviewed the connection between micronutrient deficiencies and mitochondrial disorders [35]. Avoiding nutrient deficiencies can help maintain metabolic flexibility by optimizing mitochondrial function [36]. Some mitochondrial-targeted supplements are discussed below.
**Increasing mitochondrial efficiency**Adequate nutrient supply for energy demandSufficient micronutrient supply (vitamin-based coenzymes and cofactors)Caloric restrictionModerate physical activityDelaying elevation in aerobic glycolysis with age

Precursors of coenzymes nicotinamide adenine dinucleotide (NAD+), flavin adenine dinucleotide FAD, and coenzyme Q (CoQ, CoQ10 in humans) increase the efficiency of oxidative phosphorylation. The NAD+, which includes two covalently linked “mononucleotides” (nicotinamide mononucleotide or NMN and AMP) [37], has a significant role in energy metabolism such as mitochondrial electron transport, glycolysis, and the citric acid cycle [38] to ATP [39], and is a substrate for NAD+-dependent enzymes like PARPs and sirtuins, which are involved in cellular repair and maintenance [40,41,42]. Thus, sufficient NAD+ availability generates a system with low entropy. NAD+ levels decrease with age at cellular, tissue, and organismal levels as a result of inflammation, defects in nicotinamide phosphoribosyltransferase (NAMPT) mediated NAD+ biosynthesis, and sirtuins and PARP-mediated NAD+ degradation [43,44]. NAD+ is also cleaved by nucleotide phosphatases (CD73) or glycohydrolases (CD38 and CD157) [45]. Consequently, this reduces the production of cellular energy and DNA repair and alters genomic signaling, resulting in a higher prevalence of chronic disease and ageing [46]. In addition, the NAD+/NADH ratio impacts ROS and the formation of oxidative stress by regulating the production of intracellular ATP, various metabolic enzymes, and redox status. A boost in NAD+ and/or NAD+/NADH ratio may enhance cell defense and induce DNA repair and apoptosis by activating PARPs and sirtuins [47,48]. NAD+ levels can be controlled by lifestyle and diet, e.g., fasting, caloric restriction, exercise, low glucose availability, and heat shock [41,42,43,44,45,46,47]. NAD+ levels can also be increased by food and commercial supplements [49] or NAD+ boosters, including nicotinamide mononucleotide, nicotinic acid, nicotinamide, tryptophan, and nicotinamide riboside, as reviewed in [46]. In addition, NAD+ levels may be raised by reducing NAD+ utilization by PARP enzymes or CD38/CD157 using PARP, CD38, and SAM1 inhibitors [43,50,51,52]. FAD is a covalently bound prosthetic group of electron transport chain complex II and a prosthetic group/coenzyme of some other enzymes, including those participating in fatty acid degradation (Figure 2). Complex II is a part of the electron transfer chain and Krebs cycle, and transfers two electrons from succinate through the reduction of FAD to the electron transport chain and yields fumarate in the Krebs cycle [53]. On the other hand, electrons transferred to FAD during fatty acid degradation also enter ETC through CoQ independently of complex II [53]. In this way, electrons originating from the oxidation of fatty acids via FADH_2_ enter ETC through complex II from the oxidation of acetyl-coenzyme A in the Krebs cycle and independently of complex II if the electrons are transferred to FADH_2_ during β-oxidation. By facilitating the movement of electrons through ETC and contributing to the proton gradient, FAD plays a critical role in increasing the efficiency of oxidative phosphorylation and ATP synthesis in the mitochondria [54,55]. CoQ is a vital component of the electron transport chain, which is the final step in cellular respiration. At this stage, electrons are passed through a series of protein complexes, and CoQ acts as an electron transfer agent between complexes I and III and II and III. Coenzyme Q plays a multi-faceted role in improving mitochondrial function as a part of the electron transport chain, acting as an antioxidant; it is a part of ETC super-complexes consisting of complexes I, III, and IV dedicated to the oxidation of NADH as well as interacting with complex II and other enzymes that use it as a cofactor, a so-called free pool [56]. Its role in the regulation of sulfide metabolism and as a coenzyme of mitochondrial FAD containing acyl-coenzyme A dehydrogenases and proline dehydrogenases links energy production to other metabolic pathways and needs further evaluation. At least in the animal model exposed to ultraviolet B radiation, adding CoQ elevated SOD2 and GPx [57]. Increased oxidative stress and mitochondrial efficiency decline with age may be partly due to a CoQ reduction [58,59]. Nevertheless, there is a paradox of the role of CoQ levels in ageing. CoQ supplementation can benefit animal models of disease and human studies in conditions associated with oxidative stress; however, some defects in its biosynthesis increase the lifespan in animal models, possibly due to mitohormesis [60]. Vitamins riboflavin (B2), niacin (B3), B6, B12, folic acid, pantothenic acid, and vitamin C are needed to synthesize CoQ (Table 1) [61]. Derivatives of the B vitamins thiamine, riboflavin, niacin, pantothenic, and lipoic acid are also crucial for acetyl CoA supply to the tricarboxylic acid cycle [35] as cofactors for the enzyme pyruvate dehydrogenase, a PDH complex [35,62,63,64]. The PDH complex is responsible for converting glucose-derived pyruvate to acetyl-CoA, which is an important step in the overall process of ATP production. Thiamine is also important for the function of the α-ketoglutarate dehydrogenase complex, which converts α-ketoglutarate to succinyl-CoA, reducing another NAD+ molecule and feeding more electrons to the ETC for ATP synthesis [65,66]. Riboflavin (vitamin B2) serves as a precursor for two important coenzymes, flavin mononucleotide (FMN) and FAD, which also form integral parts of several protein complexes in the ETC [67,68]. 

Cobalamin (vitamin B12) indirectly affects ATP formation and mitochondrial efficiency through its involvement in methionine metabolism, folic acid metabolism, and fatty acid metabolism, which in turn affects the efficiency of mitochondrial processes and ATP production [35,69]. Low folic acid (vitamin B9) reduces the ability to repair DNA since it participates in DNA repair through de novo DNA synthesis and methylation [70]. DNA methylation is an epigenetic modification essential for the normal regulation and development of the genome. Ageing leads to a considerable change in the distribution of 5-methylcytosine (the product of DNA methylation) throughout the genome and a reduction in global DNA methylation in the genome [71]. Selenium is included in the selenoproteins, of which some are important antioxidant enzymes. Adequate selenium uptake is necessary to maximize the activity of selenium-containing enzymes glutathione peroxidase and thioredoxin reductase. Seo et al. (2002) observed that selenium (selenomethionine) modulates base excision repair by reducing cysteines in the protein P53, which leads to better DNA excision repair [72]. Fischer et al. (2006) have also shown that selenomethionine preferentially induces the DNA repair branch of the p53 regulation. Zinc is another essential mineral that could modulate base and nucleotide excision repair [73]. Zinc is an essential component or cofactor of more than 300 mammalian proteins. It is a key essential component of DNA-binding proteins with zinc fingers and cytosolic CuZnSOD and a number of proteins involved in DNA repair [74]. Zinc deficiency leads to DNA damage [75]. L-carnitine improves mitochondrial function by transporting fatty acids into mitochondria, promoting acetyl-CoA supply and thus ATP production, reducing the accumulation of toxic metabolites, providing antioxidant support, stimulating mitochondrial biogenesis, and maintaining the integrity of the mitochondrial membrane [36,76]. MitoQ is a derivative of Coenzyme Q10. By preventing oxidative damage and maintaining mitochondrial membrane integrity, MitoQ can contribute to preventing or mitigating mitochondrial dysfunction. Dysfunctional mitochondria are less efficient at generating ATP, so maintaining proper mitochondrial function can support ATP production [77,78,79,80]. Pyrroloquinoline quinone (PQQ) could enhance both the quantity and efficiency of mitochondria. PQQ engages with cell signaling pathways and impacts the energy-associated mitochondrial metabolism [81]. PQQ offers protection against premature senescence and is linked to the SIRT1/PGC-1α signaling pathway, mitochondrial structure, and mitochondrial respiratory capacity [82]. Finally, a balanced intake of vitamins C, K and E influences the optimal function of the mitochondria by supporting the direct antioxidant defenses. 

**Table 1:** Vitamins, minerals, and nutrients required for mitochondrial energy production.

Micronutrient	Functions in Bioenergetic Mitochondrial Processes	References
Thiamine (vitamin B1)	- conversion of pyruvate to acetyl-CoA - a cofactor for the alpha-ketoglutarate dehydrogenase complex, which catalyzes the conversion of alpha-ketoglutarate to succinyl-CoA in the citric acid cycle	[35,83,84]
Riboflavin (vitamin B2)	- involved in fatty acid oxidation in the TCA cycle - an important building block for complexes I and II; CoQ synthesis - precursor of flavin adenine dinucleotide (FAD) and flavin - mononucleotide (FMN)	[35,61,67,68,83]
Niacin (vitamin B3)	- a crucial role in the electron transport chain - CoQ synthesis; acetyl-CoA supply to TCA - a precursor of nicotinamide adenine dinucleotide (NAD) and nicotinamide adenine dinucleotide phosphate (NADP)	[35,61,85]
Pantothenic acid (vitamin B5)	- a precursor for the synthesis of coenzyme A (CoA) - CoQ synthesis	[61,86]
Pyridoxine (vitamin B6)	- CoQ synthesis	[61]
Biotin (vitamin B7)	- involvement in carboxylation reactions - essential for fatty acid oxidation	[83]
Folate (vitamin B9)	- involvement in one-carbon metabolism, which is necessary for the synthesis of nucleotides, amino acids, and cellular methylation reactions - CoQ synthesis	[61,87]
Vitamin B 12	- CoQ synthesis - essential cofactor in the formation of succinyl-CoA (metabolite of the TCA cycle)	[61,88]
Lipoic acid	- a cofactor for pyruvate dehydrogenase (PDH) and alpha-ketoglutarate dehydrogenase (KGDH) complexes	[35,89]
Ascorbic acid (vitamin C)	- biosynthesis of carnitine - antioxidant - CoQ synthesis	[61,83,90]
Tocopherol (vitamin E)	- lipid soluble antioxidant in cell membranes - antioxidant defense	[91]
Vitamin K	- antioxidant defense	[91]
Selenium	- positively affects complex I and IV performance - cofactor of some antioxidant enzymes (glutathione peroxidase; glutathione reductase; thioredoxin reductase)	[92]
Zinc	- cofactor in many DNA binding proteins, DNA repair	[75]
CoQ	- antioxidant - stimulates the activity of complex I and II as electron acceptors	[93]
Carnitine	- facilitates the entry of fatty acids into the beta-oxidation pathway	[94]

In addition, vitamin C contributes to the synthesis of carnitine [95], which is essential for fatty acid metabolism in the mitochondria. Vitamin K supports mitochondrial performance by promoting the activity of the mitochondrial enzymes involved in energy production, and vitamin E indirectly modulates the fluidity of the mitochondrial membrane [96].

The equilibrium between ATP demand and nutrient supply sets both the rate of ATP synthesis and the ROS level generated by mitochondria, which will be discussed in the next paragraph.

### 2.2. Eating Habits and Their Effect on ATP Generation

Dietary habits, especially excessive or restricted diets, can have significant effects on mitochondrial efficiency. When an excessive number of calories are consumed, especially in certain nutrients such as sugars and unhealthy fats, it can lead to a state of overload or overeating. This can have several negative effects on mitochondrial efficiency, including the development of oxidative stress, disruption of the equilibrium of mitochondrial dynamics, biogenesis and mitophagy (a higher proportion of dysfunctional mitochondria), and possible insulin resistance (associated with impaired mitochondrial function and lower ATP production). On the other hand, energy restriction, such as caloric restriction or specific dietary intervention, may also affect mitochondrial efficiency by improving mitochondrial function. Three distinct processes control the efficiency of mitochondrial respiration, namely (1) ATP turnover, which is set by cellular ATP consumption, (2) substrate utilization, by the presence of fuel in the mitochondrial matrix and its oxidation to produce NADH and FADH_2_ (glycolysis, fatty acid oxidation and TCA cycle), and (3) proton leak, which is set by the basal permeability of the inner membrane to protons [97].

Maintenance of antioxidant protection and cellular repair systems consumes energy. Mechanisms that safeguard cells against the harmful effects of oxidative stress and damage (e.g., endogenous antioxidant defense, DNA repair processes) consume substantial quantities of ATP when triggered in all cellular compartments of an individual organism over an extended period of time, so their optimal induction seems impractical for preventing all oxidative damage throughout life. Kowald and Kirkwood projected that immortality could be practically attained if 55% of the overall energy of the simulated cell was used to repair and/or prevent free radical and oxidative damage [13,14]. Why was more energy not invested in the maintenance and repair processes of the body? Energy minimization is favorable from an evolutionary perspective because there is no inherent evolutionary incentive to maintain physical fitness beyond the point at which most individuals have successfully reproduced. Under normal conditions, suboptimal energy content is allocated to cell repair systems, resulting in the gradual deterioration of body structures over time (i.e., ageing and increased entropy) [15]. It may be more energetically advantageous to repair or destroy damaged biomolecules (DNA, proteins, lipids) than to invest large amounts of energy in constantly activated endogenous defense systems [98]. Therefore, energy conservation is important for controlling the intensity of defense and repair processes and thus for lowering the entropy.

#### 2.2.1. High Energy Supply and Overeating

**Figure 3:**
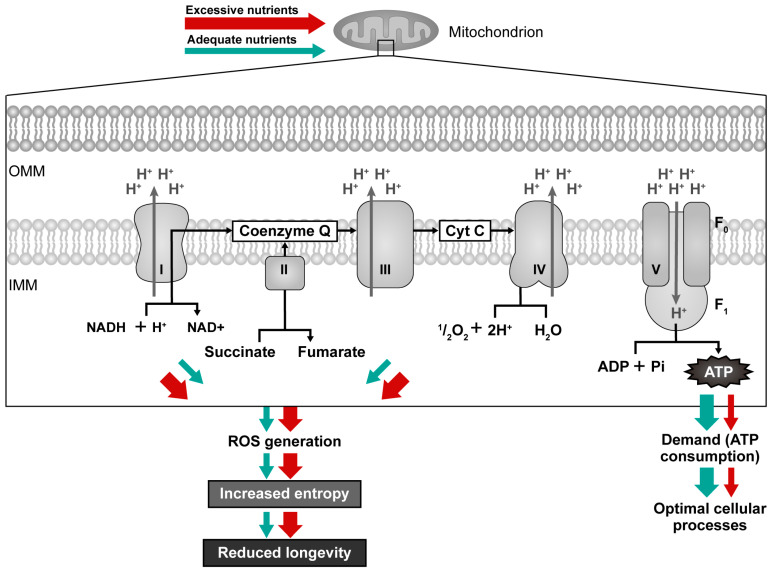
Mitochondrial efficiency with high nutrient supply/low ATP demand (red arrows) compared to adequate nutrient supply/adequate ATP demand (green arrows). Electrons escaping from the electron transport chain (ETC) generate ROS, and these molecules can damage the ETC components and mitochondrial DNA, leading to reduced ATP production and a further increase in intracellular ROS levels, resulting in further oxidative damage and a decline in mitochondrial function.

When, what, and how much we eat affects intracellular NAD+ bioavailability by modifying the transport of electrons in mitochondria. Postprandial (after a meal) oxidative stress is marked by heightened susceptibility of the organism for oxidative damage after nutrient excess, e.g., consuming a meal that contains many lipids and/or carbohydrates [99]. If the ATP requirement is low, no ATP is consumed, and the ATP synthase reduces its activity as part of the cell’s regulatory response to maintain energy balance, which also causes a change in the balance of ions across the membrane [100]. As respiratory complexes cannot pump protons against an electrochemical gradient that is too high (overloaded), the electron transfer slows down; NADH oxidation is affected because the flow of electrons from NADH to the ETC is impeded, NADH cannot be oxidized, and the entire Krebs cycle and oxidative phosphorylation also slow down [101,102,103]. A combination of high NADH levels and an overloaded membrane potential causes electrons to escape from the respiratory chain and react directly with oxygen, forming ROS [47,48] that promote the reverse Krebs cycle and the epigenetic shift associated with growth and inflammation [104,105]. Specifically, when an individual frequently overeats, higher quantities of energy become available, and mitochondria tend to generate more ROS (Figure 3—red arrows) [106,107]. For instance, a sedentary lifestyle, excessive consumption of food, and over-intake of fat and sugar have been related to a downregulation of NAMPT gene expression [108], leading to the accumulation of NADH. Following a substantial meal, large quantities of electrons from sugars enter the mitochondria generating more superoxide at complex I (NADH: ubiquinone oxidoreductase) and complex III (ubiquinol: cytochrome c oxidoreductase) [109]. Efficient flow of electrons and prevention of electron leakage (superoxide formation) may be reached if more ATP is regularly used, e.g., through moderate exercise or some other type of physical activity, as this raises the AMP/ATP ratio and NAD+ availability [110,111,112]. The mitochondrial electron transport progresses at a rate matching the extraction of energy from the proton gradient. The movement of electrons is propelled by the free energy on disposal from energy carriers. It is limited by the chemiosmotic gradient. The equilibrium between ATP demand and the supply of nutrients dictates both the rate of ATP synthesis and the level of ROS generated by mitochondria [97]. Thus, ROS production decreases with high ATP demand. A diet high in sugar and fat leads to an overload of energy, resulting in a lowered NAD+/NADH ratio [110,113,114]. It can also cause increased blood glucose and insulin levels and enhanced production of ROS, triggering postprandial oxidative stress and oxidative damage [115,116]. For this reason, work rather than rest should be performed after eating a large portion of food (nutrient overload). 

High nutrient intake and lower energy requirements cause inhibition of mitochondrial fusion, leading to fragmentation and lower mitochondrial coupling (efficiency), which is associated with heat generation and increased autophagy [117,118]. Autophagy has a critical role in eliminating damaged or nonfunctional mitochondria through a selective process known as mitophagy. When autophagy eliminates redundant or dysfunctional molecules or organelles, it enables systematic recycling of cellular components [119,120], thus decreasing entropy and increasing order. Excessive nutrient uptake can hinder autophagic flux by inhibiting the lysosomal degradation and thus damaged mitochondria [118], resulting in the accumulation of dysfunctional mitochondria. In contrast, bioenergetic adaptation during starvation homogenizes the mitochondrial community and prevents the creation of the pre-autophagic pool [97], thus increasing order.

#### 2.2.2. Low Nutritional Load and Energy Restriction

Adaptation to food deprivation may promote longevity; animals that are food-deprived increase their investments in cellular maintenance functions. It seems that these adaptive mechanisms/pathways evolved at the early stages of evolution and are also conserved in higher animals, including humans [121]. Limiting calorie intake appears to be the most successful non-genetic approach for extending lifespan. Research across various species has shown that decreasing caloric intake about 30% below *ad libitum* levels of a nutritious diet can extend life-span, lower the occurrence of age-related diseases and postpone their onset, as well as influence the regulation of excess glucose, cholesterol, triglycerides, and LDL as observed in caloric restricted humans [122,123]. Low nutrient loading leads to a reduction in the production of mitochondrial free radicals (Figure 3) [107]. Energy restriction triggers a metabolic shift that results in a more effective transport of electrons in the mitochondrial respiratory chain [124]. More efficient transport of electrons can result in mitochondria producing less ROS. This is due to the decreased loss of electrons within the respiratory chain [125,126]. Long-term CR resulted in a 45% reduction in mitochondrial H_2_O_2_ formation, a 30% decrease in oxidative damage to mtDNA in the hearts of rats [127], a 28% decrease in mitochondrial ROS formation, and a 30% fall in oxidative damage to mtDNA in the skeletal muscle of rats [128]. Furthermore, CR improves stress resistance [129] and genomic stability [130]. At first, there seems to be a paradox regarding nutrient intake, cellular energy, and damage repair. On one hand, nutrients provide sufficient energy (ATP) to run processes that repair molecular damage. On the other hand, many studies confirmed that excessive nutrients and obesity accelerate ageing, whereas CR increases life-span [131]. While CR animals generate fewer free radicals, their metabolic rate (oxygen consumption per gram of tissue) remains unchanged. Paradoxically, CR increases mitochondrial respiration efficiency and elevates rather than lowers the metabolic rate [132,133]. The shift from glucose to ketone bodies during CR causes numerous alterations in gene expression and metabolic adaptations. Increased β-hydroxybutyrate (βOHB) production by CR, blocks the activity of histone deacetylases and activates forkhead transcription factor O subfamily member 3a (Foxo3a) and metallothionein 2 (Mt2) [134]. While genes for glucose metabolism and glycolysis are downregulated, respiration genes are upregulated [135,136]. Additionally, ketone metabolism lowers the generation of ROS in normal cells [137] by oxidizing coenzyme Q and reducing NADP+ as well as increasing the reduced form of NAD+ and glutathione [138,139,140] thus decreasing free radical damage [141]. NADPH in its reduced form is the donor of reductive potential to glutathione and thioredoxins, which can be used by glutaredoxins, peroxiredoxins, and glutathione peroxidases to quench ROS [142]. 

Thus, CR does not function by slowing the metabolism and consequently impeding the generation of free radicals. In contrast, it seems that CR results in life extension since it boosts respiration and related downstream phenomena. Thus, the primary factor contributing to a decrease in oxidative stress and damage in animals that are subjected to CR seems to be linked to a decrease in the production of mitochondrial free radicals, as CR causes a metabolic shift resulting in more efficient electron transport through the mitochondrial respiratory chain [124] and improved efficiency of damage repair system(s). Consequently, intracellular entropy is decreased as the reductions in oxidative damage by CR lead to less ROS formation [143,144]. Indeed, increased DNA repair in CR animals was shown in many studies. Cabelof and colleagues (2003) observed that CR enhances genomic stability by elevating DNA repair by base excision repair [145]. CR also attenuates the protein turnover decrease [118,119]; oxidatively modified proteins are removed more efficiently by increased proteasome activity [146]. During ageing, a decrease in the abundance of proteins involved in mitochondrial function, electron transport chain, citric acid cycle and fatty acid metabolism was observed, as well as an increase in the abundance of proteins involved in glycolysis and oxidative stress response. This proteome remodeling can be significantly reversed by short-term CR [147]. CR also improves DNA repair through base excision and non-homologous end joining [148]. Comparable impacts have not been identified for mitochondrial DNA [149]; enhanced genomic stability of mitochondrial DNA seems to be a consequence of decreased ROS production by mitochondria [150]. Three key players affected by energy restriction are deacetylases sirtuins, FOXO transcription factors, and cell cycle regulatory proteins [151,152,153,154]. Energy restriction can activate sirtuins and promote their beneficial effects, including improved mitochondrial function and enhanced cellular stress response. Activation of FOXO by sirtuins can stimulate the expression of genes associated with stress resistance, DNA repair, apoptosis, metabolic adaptations, and longevity. FOXO proteins can interact with cell cycle regulators to arrest the cell cycle and inhibit cell proliferation by upregulating cyclin-dependent kinase inhibitors (CDKIs) [155,156]. CR influences CDKI expression levels in a context-dependent manner since liver regeneration was faster after partial hepatectomy in CR-restricted mice [157]. Energy restriction contributes to maintaining cellular integrity, reduction of cellular damage accumulation, and entropy reduction.

#### 2.2.3. Morphology Changes of Mitochondria—How Do Mitochondria Respond to Cellular Energy Levels?

The balanced process of mitochondrial fusion and fission is critical for maintaining a healthy mitochondrial network and cellular energy homeostasis. Alterations in mitochondrial dynamics and architecture (mitochondrial fusion, fission, and mitophagy) are a mechanism for bioenergetic adjustment to metabolic demands by regulating bioenergetic efficiency and energy consumption. Alterations in mitochondrial structure mediated by nutrients and their metabolites signify an adjustment to alterations in the ATP demand and supply [97] and activation of quality control [158]. In general, conversion toward fusion protects against cell death, and a shift toward fission increases sensitivity to apoptosis [159,160]. Mitochondrial fission is important for separating damaged areas of a mitochondrion and helps promote mitophagy. By dividing into smaller mitochondria, the damaged portion can be isolated and targeted for degradation. Mitophagy thus enables the cell to selectively eliminate damaged mitochondria, while sparing the healthy ones. Adaptation to an oversupply of nutrients causes mitochondria in a separated state (smaller, fragmented mitochondria) in contrast to the longer mitochondria in the starved cells [117,161]. Mitochondrial elongation is related to conditions that require elevated ATP synthesis capacity, so mitochondria are fused during prolonged fasting or caloric restriction to increase their efficiency in producing energy from available nutrients [97]. Low nutrient loading causes acute suppression of mitochondrial fission [161,162], increases the number of mitochondrial cristae linked to ATP synthase dimerization, and consequently increases ATP synthesis activity [161]. In contrast, decreased fusion or increased fission results in the shortening of mitochondria in states of decreased bioenergetic efficiency (excessive nutrient availability) [97]. Coordination between mitochondrial fusion, fission, and autophagy is critical for maintaining cells’ healthy and functional mitochondrial pool. Misregulation of any of these processes can lead to the accumulation of damaged mitochondria, which is associated with increased entropy and various diseases and ageing.

### 2.3. Physical Activity, Exercise, and Mitochondrial Function

Moderate physical activity increases mitochondrial efficiency and, subsequently, the formation of ROS, ultimately triggering an adaptive response that leads to increased maintenance and repair, culminating in metabolic health and prolonged longevity. Both low energy status and increased ROS during exercise can influence various cellular signaling pathways and positively influence the expression of several genes in eukaryotic cells, including antioxidant enzymes, stress proteins, DNA repair proteins, and mitochondrial electron transport proteins [163]. Exercise may promote longevity through pathways common to previously discussed effects of CR. During intense exercise, ATP is consumed; this increases the demand for NADH as an electron donor, which ultimately leads to increased formation of oxidized NAD+ and a decrease in NADH, i.e., an improved NAD+/NADH ratio. The total amount of NAD+ is not considerably altered during the redox reaction; however, the NAD+/NADH ratio is altered for the benefit of NAD+ [164]. Engaging in exercise and aerobic physical activities boosts NAD+ levels, partly because it stimulates the expression of NAMPT in skeletal muscles [165]. It counteracts the natural decrease in NAD+ that occurs with age by promoting the NAD+ salvage pathway [166] via the 5′ AMP-activated protein kinase (AMPK) pathway [108]; this has been demonstrated in both rodent and human research [165,167,168], where the SIRT1 activity and mitochondrial function were enhanced [169,170,171]. Exercise also stimulates the production of various signaling molecules, such as PGC-1α (peroxisome proliferator-activated receptor gamma coactivator 1-alpha), which significantly contributes to activating genes involved in mitochondrial biogenesis [172,173]. Improving the NAD+/NADH ratio by increased ATP consumption (e.g., exercise) or more efficient ATP production (e.g., intermittent fasting, consumption of small food portions, and CR) regulates the extent of superoxide formation from the transfer of electrons to molecular oxygen at mitochondrial complexes I and III and may therefore reduce the intensity of oxidative damage [174]. The increased energy requirements during exercise trigger AMPK activation, which has the capability to modulate NAD+ bioavailability, making it also available for sirtuins and PARPs activation [175], thus decreasing entropy.

### 2.4. Shifting Aerobic Glycolysis to Oxidative Phosphorylation

The pathways linking the age-related increase in glycolytic flux to mitochondrial dysfunction are not yet fully understood. Alterations in cellular metabolism that lead to elevated glycolytic fluxes and hyperglycemia serve as origins of increased entropy as a consequence of cellular damage since most glycolytic intermediates indirectly promote advanced glycation end product (AGE) formation and ROS-induced damage [176]. Marin and Sabater (2017) [177] calculated about 10% lower entropy by fermentation than respiration in human cancer tissues (at 5 mM glucose, 2.9 mM lactate, pH 7.4, (380 ppm = 38 Pa) CO_2_, and 37 °C/310 K) in human tissues. However, the authors also reported an inverse situation, namely that entropy is lower by respiration when the CO_2_ concentration increases above 380 ppm. It is conceivable that the CO_2_ concentration can increase locally in mitochondria near the site of oxidative phosphorylation (respiration) because of the proximity of the site of CO_2_ production. Also, apart from glucose, fatty acids, proteins, and other molecules are used for respiration and would need to be accommodated in the entropy of respiration calculations. Finally, the thermodynamic efficiency of mitochondrial respiratory complexes (I, III, and IV) varies from 80–90% during oxidative phosphorylation and can approach zero at the resting steady-state level when there is a high concentration of ATP compared to those of ADP and Pi [178]. Therefore, the optimal functioning of respiratory complexes and mitochondria is crucial for health and the maintenance of low entropy.

Elevated aerobic glycolysis (fermentation) in older age could be a cellular strategy to minimize ROS production by less efficient mitochondria or compensation for lower mitochondrial function by higher glycolytic activity [59]. The degree of oxidative damage caused by mitochondrial dysfunction increases steadily with age [124,179]. Because mitochondrial function maintains the differentiated state, cells that upregulate fermentation for survival are at increased risk of becoming less differentiated [180]. Impaired respiration and energy metabolism precede and underlie genome instability because cellular repair systems are supplied with less energy [180]. For example, mitochondrial dysfunction downregulates the expression of the apurinic/apyrimidinic endonuclease APE1, which regulates DNA transcription and repair [181,182]. The decrease in NAD+ levels associated with ageing results in impaired mitochondrial function, ultimately contributing to the occurrence of the Warburg effect (aerobic glycolysis) [183], described as a rise in the rate of glucose uptake and preferential generation of lactate, even when oxygen is available [184]. Another reason for increased aerobic glycolysis is ROS-induced damage to the respiratory chain that encourages a hypoxia-like state [185], stabilizes the transcription factor HIF, and upregulates glucose transporters. Decreased NAD+ availability alters mitochondrial activity and is connected to elevated ROS production [186], lower oxidative metabolism, and mitochondrial biogenesis. Over time, the gradual deterioration of mitochondrial function and the breakdown of oxidative phosphorylation (OXPHOS) trigger a transition in metabolism. This transition involves a shift from relying on ATP generated within the mitochondria to a greater dependence on glycolysis. This shift occurs due to the buildup of hypoxia-inducible factor 1 alpha (HIF-1α), which metabolically reprograms the fate of intracellular glucose [187,188], and decreases the activity of respiratory complexes I, III and IV through expression of specific genes [183]. These processes result in the Warburg effect, contributing to the development of metabolic syndrome and the onset of various degenerative diseases [105,183,189,190]. Furthermore, the elevated levels of intracellular NADH also hinder OXPHOS by stimulating pyruvate to lactate conversion and reducing the permeability of the voltage-gated anion channel in the outer mitochondrial membrane [191]. The hypothesis presented by Poljsak et al. (2019) [105] states that when cells are deficient in NAD+ (e.g., increased NAD+ consumption due to increased cell activity or increased metabolic demand, increased DNA repair and sirtuin activity, increased oxidative stress and increased need for NAD+-dependent antioxidant systems, and disruption of NAD+ biosynthetic pathways due to a lack of NAD+ precursors), they initiate the program to modulate the Krebs cycle and the electron transport chain. Glycolysis produces only two mols of ATP per one mol of glucose, while oxidative phosphorylation produces around 36 mols of ATP per mol of glucose [192]. Activated fermentation allows such a cell to enter its primitive state, possibly resulting in an elevated entropy state within the cell. Normal cells expend significant energy to maintain a low entropy state. Considering the locality of the second law of thermodynamics, entropy can be divided into entropy derived from a chemical reaction and entropy produced by the diffusion of signaling molecules [193,194]. Organisms employ both information and energy to sustain a stable entropy that is far from thermodynamic equilibrium; high entropy implies high uncertainty or disorder in the system, meaning that more information is needed to describe it accurately [195]. Energy losses from compromised mitochondria limit the energy invested to repair damage and genomic stability, and may increase entropy over time, as it influences the cellular organization [105]. A metabolic switch to aerobic glycolysis could result from a pre-historic (re)program. Prior to the existence of oxygen in the atmosphere, the primary phenotype and default state of metazoan cells were characterized by proliferation and fermentation [196].

### 2.5. Hormesis—Moderate Exposure to Stressors Activates Damage Repair Systems and Reduces Entropy

According to Sies [30], mechanisms for the maintenance of homeostasis can be divided into reactive (feedback, counter-regulation) and predictive (feedforward, anticipatory) modes. For the latter, the capability of preconditioning in response to endogenous and exogenous cues is important for adaptive stress responses and hormesis. Toxicologists use the term “hormesis” to describe a biphasic dose response to an environmental agent where low doses have a positive effect while high doses lead to toxicity [197]. Various stressors result in distinct cellular responses: activated cell repair mechanisms, temporary adaptation to some stressors, autophagy induction, or initiated cell death. UV radiation, heat shock, certain phytochemicals, ischemia, exercise, and dietary energy restriction trigger adaptive responses that modulate repair processes and promote health in organisms ranging from bacteria to humans [198,199,200]. The nature and intensity of the stressor determine the nature of the stress response. Paradoxically, the efficiency of defence and repair can be enhanced following moderate exposure to ROS as the expression of antioxidants and several DNA and other repair enzymes is upregulated in response to moderate oxidative or other forms of stress [77,167,168]. Several proven components of a heart-healthy lifestyle, including polyunsaturated fats, physical activity, and moderate alcohol consumption can produce moderate amounts of oxidants in the body [201]. Finkel and Holbrook (2000) [202] observed that the most effective approach to boost endogenous antioxidant levels can involve exposure to oxidative stress itself, based on the classical physiological concept of hormesis. Mechanisms of the stress response include activation of cellular signaling pathways, activation of transcription factors, and cell cycle disruption to enable the repair of DNA and other damaged molecules, resulting in lower entropy. An alternative approach to mitigating ROS-induced stress/damage is to trigger an adaptive stress response to increase the body’s antioxidant and damage repair processes. Moderate stress triggered by caloric restriction, physical activity, or mimetic compounds can activate the endogenous antioxidant defence and cellular repair processes. There is constant ROS formation and some oxidative damage in the human body. This necessitates a second category of antioxidant defence mechanisms to eliminate and restore damaged biomolecules before they accumulate to result in metabolic alternations and irreversible damage [203]. Many important repair systems become inadequate in ageing, so entropy and cellular damage increase over time [204,205]. Moderate stress inducers (oxidants) or mimetics that stimulate a moderate stress response to strengthen cellular repair and maintenance systems can contribute to decreased accumulation of cellular damage. Upon perception of various types of stress, intracellular adaptive responses activate several energy-consuming (anabolic) processes and delay cell division while energy is used for cell repair and maintenance. At the molecular level, cellular stress response pathways are governed by various highly conserved signaling molecules and transcriptional regulators. These include proteins associated with insulin/insulin-like growth factor (IGF) signaling, sirtuins, mammalian target of rapamycin (mTOR), and AMPK pathways [206]. These adaptive pathways frequently include a coordinated regulation of protein synthesis and turnover, autophagy, and mitochondrial function [207]. 

### 2.6. Signaling Pathways Important for Cellular Damage Repair and Cellular Energy Signaling

Several signaling pathways are crucial for cellular energy signaling and damage repair. The signaling network that reacts to nutritional conditions and manages growth, resistance to stress, and the ageing process includes insulin/IGF-1, sirtuin, AMPK, and mTOR [208,209]. Significant elements of the main longevity pathways include insulin/IGF-1 receptor signaling and the FOXO family of transcription factors, whose activity is regulated in part by lysine acetylation and the action of “sirtuin” NAD+-dependent deacetylases [210]. Sirtuins are a class of proteins involved in the regulation of various cellular processes, including DNA repair, gene expression, metabolism, and stress response. Sirtuins, such as SIRT1, require NAD+ as a cofactor to exert their effects. FOXO is a family of transcription factors that are crucial for the regulation of cellular processes such as DNA repair, apoptosis (cell death), cell cycle regulation, and stress responses. FOXO proteins are also included in regulating longevity and the ageing process [211]. The link between NAD+, sirtuins, and FOXO is that the NAD+ level affects the activity of SIRT1. This, in turn, may influence the function of FOXO proteins. AMPK is the key regulator of cellular energy homeostasis. It is activated when cellular energy levels are low, indicated by an increased AMP-to-ATP ratio. It has an important role in several cellular processes, including mitogenesis, PGC-1α regulation, and autophagy. AMPK activation promotes energy conservation and production by increasing glucose uptake, fatty acid oxidation, mitochondrial biogenesis, and oxidative metabolism. Mitochondrial biogenesis and oxidative metabolism are controlled by PGC-1α. AMPK activation can trigger autophagy in response to cellular stress, such as nutrient or energy deficiency, namely, AMPK phosphorylates, and activates the ULK1 complex, an important initiator of autophagy [180,181]. AMPK is activated in response to various stress signals that lead to increased AMP or ADP in the cells [212,213]. It is allosterically activated by 5′-AMP and allosterically inhibited by creatine phosphate [212,214]. The AMPK cascade is activated by metabolic stresses that either inhibit ATP production (e.g., hypoxia, hypoglycemia) or increase ATP consumption (e.g., intense muscle contraction) [215] to initiate ATP-generating pathways (such as fatty acid oxidation and glycolysis) while deactivating ATP-consuming pathways (such as lipogenesis). This signaling occurs through the phosphorylation of regulatory proteins and gene expression (long-term effects) [216,217]. The induction of AMPK suppresses the mTOR [218], a serine/threonine protein kinase that is an important regulator of cell growth, proliferation, and metabolism in response to nutrients (e.g., glucose, amino acids), growth factors (e.g., increased insulin, IGF-1, and platelet-derived growth factor (PDGF)), and cell energy status (ATP) [219]. It also has a key role in regulating autophagy, as active mTOR signaling inhibits autophagy to ensure that cellular resources are directed to growth-promoting processes rather than recycled through autophagy. When developmental growth is complete, mTOR also drives ageing, manifested by increased cellular functions and redirecting energy from repair and maintenance mechanisms to growth, thus altering homeostasis, and leading to increased age-related disease and entropy [131]. Then, any alteration that results in more molecules, or a larger body volume/mass results in an increase in entropy as more errors accumulate in the genome as cells replicate.

Elevated blood concentrations of insulin, hyperinsulinemia, lead to a decrease in insulin-like growth factor-binding proteins and stimulate the synthesis and activity of IGF-I. IGF-I regulates ageing and cell growth based on the energy and nutrients available from both the diet and the body’s reserves [220]. On the contrary, the reduction of insulin receptors as well as insulin receptor substrates below a certain threshold level contributes to the longevity of many tested organisms like worms, flies, and mice [209]. Reduced amounts of insulin-like peptides extend the lifespan of nematodes, flies, and rodents. In the case of nematodes and flies, secondary hormones downstream of insulin-like signaling appear to control the ageing process. CR reduces the levels of insulin and IGF-1 and also increases insulin sensitivity in rodents [221] and in monkeys [222].

## 3. Conclusions

Cells are inherently equipped with various maintenance, repair, and protection mechanisms that depend on their ability to generate energy. Mitochondria are central to these processes as the main generators of cellular energy. Ageing is associated with greater entropy and less energy for maintenance, repair, defence, and useful work. Increased intracellular entropy is associated with decreased efficiency of energy production resulting in a vicious cycle leading to a more disordered state and ageing. The current literature in the field of entropy and ageing poses significant challenges to conducting a comprehensive study or meta-analysis. Research-based publications that explicitly address this topic are limited. Therefore, our manuscript takes a hypothesis-driven approach to propose new directions related to entropy and ageing.

By focusing on advancing the theoretical framework and highlighting gaps in knowledge, we aim to stimulate further empirical research and contribute to the growth of this field of science. Much more detailed, systematic, rigorous and methodological studies and a more mathematized approach are desirable for the scientific discourse on entropy and ageing in the future.

This paper presents certain strategies that can indirectly impact ageing by mitigating entropy and promoting cellular health. There is no single intervention or pharmaceutical “magic bullet” to restore a low-entropy state and maintain a healthy state. Therefore, healthy lifestyle factors that include regular exercise and a balanced diet to obtain the necessary nutrients are the safe options that were verified throughout evolution.

## Abbreviation

ATP	adenosine triphosphate
AcCoA	acetyl coenzyme A
AGE	advanced glycation end products
AMP	adenosine monophosphate
AMPK	AMP-activated protein kinase
βOHB	β-hydroxybutyrate
CDKI	cyclin-dependent kinase inhibitors
CoQ	coenzyme Q
CR	caloric restriction
ETC	electron transport chain
FAD	flavin adenine dinucleotide (oxidized form)
FADH_2_	flavin adenine dinucleotide (reduced form)
FMN	flavin mononucleotide
FAD	flavin adenine dinucleotide
FOXO	forkhead transcription factor O
HIF-1α	hypoxia-inducible factor 1 alpha
IGF	insulin/insulin-like growth factor
Mt2	metallothionein 2
mTOR	mammalian target of rapamycin
NAD+	nicotinamide adenine dinucleotide (oxidized form)
NADH	nicotinamide adenine dinucleotide (reduced form)
NAMPT	nicotinamide phosphoribosyltransferase
NMN	nicotinamide mononucleotide
O_2_	molecular oxygen
OXPHOS	oxidative phosphorylation
PARP	poly (ADP-ribose) polymerase
PGC-1α	peroxisome proliferator-activated receptor gamma coactivator 1-alpha
Pi	inorganic phosphate
PQQ	pyrroloquinoline quinone
ROS	reactive oxygen species
